# Chronic Catarrh

**Published:** 1885-05-20

**Authors:** Geo. W. Groff

**Affiliations:** Lewisburg, Pennsylvania


### CHRONIC CATARRH.

By Geo. W. Groff, M. D., Lewisburg, Pennsylvania.

This disease is so common in the Eastern parts of the United States that it may be considered well nigh universal, and it is also said to be common in most parts of the world. No age is exempt, unless it be children for the first few weeks after birth, but in a little while they become much subject to catarrhs and colds.

### THE SYMPTOMS.

These are "similar to those of coryza (cold in the head), but less acute ; discharge from the nose, either through the nostrils or throat ; formation of greenish scales or scabs in the nose ; mucous membrane swollen, often obstructing breathing ; in some cases, diminished secretion, constituting 'dry catarrh ;' often offensive breath."

As people at large understand it, chronic catarrh may exist in the nasal passages, in the pharynx and Eustachian tubes, in the larynx, or in the bronchial tubes. A chronic inflammation of any part of the respiratory passages, accompanied by the discharge of hardened mucus and unpleasant breath, constitutes "catarrh."

### CAUSES.

For many cases of catarrh, no cause can be assigned. Some think the causes are generally obscure, but the following may safely be named :

1. Neglect of common "cold in the head," which often passes into chronic catarrh.

2. Insufficient clothing.
3. Sudden and great climatic changes.
4. Errors in diet; especially the excessive use of fats, pastries, and sugars by those of sedentary habits.
5. An insufficiency of fresh air, sunshine, and outdoor physical exercise.
6. A scrofulous, gouty, or syphilitic taint in the blood.
7. Torpidity of the liver. It is probable that the liver is nearly always in a state of incomplete activity in cases of chronic nasal catarrh. The hardened discharges from the nose are said to contain a considerable amount of *cholesterine*, a substance peculiar to the bile.
8. Foreign particles, as dust, irritating gases, dead bone, or possibly a tumor growing in the passages.

### EXTENSIONS OF THE DISEASE.

Catarrh may be located in the mucous membranes of the head for a long while, and produce no special inconvenience, but the inflammation frequently extends from the first seat to deeper and more inaccessible parts.

1. It may extend to the bones of the nose, and these may be completely destroyed. It is when the bones of the head are affected that the breath becomes so horribly offensive. It is very seldom that catarrh attacks the bones unless there is a syphilitic taint in the blood, or possibly only a scrofulous taint, in rare cases.
2. It may extend into the *frontal sinus*, a cavity in the bones, above and between the eyes, when it causes a persistent dull aching in this part of the head.
3. At other times it extends into the antrum of Highmore, causing dull, heavy aching in the face.
4. It not infrequently enters and extends throughout the course of the Eustachian tubes to the middle ear, and is a common cause of deafness.
5. The disease often extends backward and downward from the nasal cavity to the pharynx, causing sore throat or pharyngitis; thence it may continue downward to the larynx, where it attacks the vocal cords. In some cases, it is possible that catarrh may commence in the pharynx or larynx and extend upward to the head and downward to the lungs. This is said to be true in children.
6. The inflammation may continue to descend from the larynx until, reaching the bronchical tubes, chronic bronchitis is produced, and finally entering the minute bronchioles, and ultimate air cells, *consumption* may result. It is probable that not a few cases of neglected nasal catarrh end in pulmonary consumption.

### THE DANGERS.

Although catarrh may exist a long time, producing no evident evil effects, it should never be neglected, for, when left a long time, it becomes deep seated, and one of the most difficult of diseases to cure. It very often makes the breath intensely foul. Entering the Eustachian tubes, it becomes one of the common causes of deafness. It may produce most painful and dangerous abscesses in the frontal sinus and in the antrum of Highmore. It may produce a chronic sore throat, destroy the voice, or much injure it. It often causes an incurable bronchitis, or may finally end in consumption. In young children catarrh tends rapidly to reach the lungs. Certainly there is reason enough to attend to it early.

### GENERAL TREATMENT.

The body should always be warmly clad, best with woollen clothing next to the skin. Climatic changes are to be guarded against by proper adjustments of clothing. Draughts and all causes known to produce colds are, so far as possible, to be avoided. Baths are to be *freely* employed, to excite the skin to activity. Rubbing the body with a coarse towel, and with the hands, and carefully kneading of the bowels, is beneficial. Pure water should be used as a drink in large quantities, but tea, coffee, spices and condiments sparingly, while tobacco and alcohol should be banished. The patient should be in the open air and in the sunshine as much as possible; a number of hours daily will prove most beneficial. Physical exercise is all important in the cure of this disease. This should be in amount sufficient to thoroughly warm the whole body. It must be regularly and systematically employed. There are no forms of exercise better than gardening, sawing wood, walking in the country, or some mechanical occupation, as carpenter work. Rich foods and pastries are to be avoided, as is an excess of all food. Much butter, fatty foods, sugars and animal foods must be avoided. The diet should be simple, largely of vegetable foods, but the *body must be well nourished.* Whatever tends to make the liver torpid must be avoided. Tonics and, in general, measures calculated to give vigor to the system will be likely to prove useful. Among these must not be forgotten country air, sea bathing, long journey, and a sea voyage.

An excellent tonic for those of a scrofulous diathesis, suffering from catarrh, is the following :

| | | |
|---|---|---|
| ℞. | Tinct. chloride iron........................... | f ℥ ss. |
| | Sulphate of quinine.......................... | ʒ ss. |
| | Glycerin......................................... | f ℥ iv. |

Dose, a teaspoonful in a wineglass of water three times a day, before meals.

Infusion of wild cherry bark and infusion of catechu, in tablespoonful doses, are also recommended.

### LOCAL TREATMENT.

The simplest form of local treatment is the drawing up into the nostrils of some medicated liquid. One of the best of these is a solution of common salt in soft water. Place a little in the hollow of the hand, and by placing this at the nostril and making a sudden, forced inhalation, it may be drawn to the seat of disease.

But it frequently happens in cases of long standing disease that the inflamed spots are so covered up with hardened mucus that no medicine can avail until the passages are thoroughly cleansed. This is best done by passing from a nasal douche copious currents of warm water into one nostril, allowing it to flow from the other. Two or three gallons of water will not be too much for this purpose. [The nasal douche should be used with great caution, being placed but very little higher than the head, when stooping over, in order not to have a strong pressure of water. The patient should also be very careful to keep the mouth open, breathe slowly, and avoid strangling. If these precautions are neglected, there is great danger that water may be forced into the Eustachian tubes, causing strangling and often rupture of the drum of the ear. Cases of this sort are known to the writer, who much prefers to use an atomizing apparatus in place of the nasal douche, although it must be admitted that the latter is more quickly efficient.—*Ed. Druggists Circular*.

In some very bad cases, water alone will not remove the hardened accumulations, when an alkaline solution may be used with success. Two or three gallons may be necessary, made in the following proportions :

| | |
|---|---|
| Soft water | 1 quart. |
| Soda or saleratus | 1 teaspoonful. |

After the passages have been thoroughly cleansed by the warm water or the alkaline solution, one of the following solutions may be used, also with the douche :

| | |
|---|---|
| Zinc sulphate | 10 grs. |
| Soft water | 1 qt. |

Or:

| | |
|---|---|
| Carbolic acid | 50 grs. |
| Soft water | 1 pt. |

Or, in cases where the fetor is very bad :

| | |
|---|---|
| Carbolic acid | 50 grs. |
| Tincture of iodine | 10 drops. |
| Soft water | 1 pt. |

All medicated solutions may be made stronger or diluted, according to the needs of each case. In all cases before making an application to the diseased membranes, the passages should be thoroughly washed out with at least a gallon of lukewarm water. Then from a pint to a quart of the medicated solution may be used. When treatment is commenced, it should be repeated three or four times the first day, after that one in twenty-four hours will answer, preferably in the morning. If the disease is in the upper part of the Schneideran membrane, the liquid can be forced up there, by compressing for a moment the nostril from which the water is flowing.

In addition to the solutions mentioned above, chlorinated soda, a tablespoonful to a pint of water, is highly commended, also a strong decoction of coffee.

Creosote in solution, the black and yellow washes, and the powders of borax, boracic acid, and of chlorate of potassium are all recommended. The powders are blown into the nostrils from a quill.

If there is a foreign body, a dead bone, or a polypus in the nose, no cure can be expected until its removal. In all cases, the treatment must be long continued.

### CHRONIC SORE THROAT.

Medicated substances are applied to the throat by gargling or by spraying. The following are good gargles :

Carbolic acid .................................. 5 grs.
Water.......................................... 1 oz.

Or :

Permanganate potass.......................... 30 grs.
Water.......................................... 5 oz.

For spraying, the following are recommended :

℞. Potass. chlorate.............................. ʒ ss.

Fl. ext. bellad.................................. f ʒ j.

Glycerin........................................ f ℥ j.

Water........................................... f ℥ iij.

M. To be applied four or five times a day.

Or :

℞. Tinct. myrrh.................................. f ℥ ss.

Tinct. hydrastis................................. f ℥ j.

Water........................................... f ℥ iv.

M. The mixture to be well shaken, and applied four or five times a day.

Local treatment must be long continued. In some cases it may be necessary to have a strong solution of silver nitrate applied.

When the disease has passed down into the larynx and trachea, great relief is derived from the inhalation of steam and other vapors. The following is good :

℞. Comp. tinct. iodine.......................... f ℥ ss.

Ammonia water.................................. f ʒ j.

Brandy.......................................... f ℥ j.

M. Shake well before using.

Place in a wide mouthed bottle, and warm with the hands, a spirit lamp, or by introducing a piece of hot wire. Place the mouth to the bottle, and inhale. The steam atomizer may be used with much success, with carbolic acid, or any other reliable disinfectant.

In all cases where catarrh tends to become chronic, a reliable medical man should be consulted.—*Drug. Gazette.*

---

**Syncope from the Use of Cocaine.**—Dujardin-Beaumetz (*Ibid.*) has observed several cases in which the hypodermic injection of weak solutions of cocaine was followed by syncope of brief duration. He attributes the occurrence to cerebral anæmia, and says that it never takes place if the patient is kept in the prone posture.—*New York Medical Journal.*

